# COMPLICATIONS RELATED TO GASTRIC BYPASS PERFORMED WITH DIFFERENT GASTROJEJUNAL DIAMETERS

**DOI:** 10.1590/0102-6720201600S10004

**Published:** 2016

**Authors:** José SAMPAIO-NETO, Alcides José BRANCO-FILHO, Luis Sérgio NASSIF, Anne Caroline BROSKA, Douglas Jun KAMEI, André Thá NASSIF

**Affiliations:** Bariatric Surgery and Metabolic Service of Holy House Hospital of Curitiba, Curitiba PR, Brazil.

**Keywords:** Anastomosis, Roux-en-Y, Bariatric surgery, Gastric bypass, Stenosis

## Abstract

**Background:**

Among the options for surgical treatment of obesity, the most widely used has been the Roux-en-Y gastric bypass. The gastrojejunal anastomosis can be accomplished in two ways: handsewn or using circular and linear stapled. The complications can be divided in early and late.

**Aim:**

To compare the incidence of early complications related with the handsewn gastrojejunal anastomosis in gastric bypass using Fouchet catheter with different diameters.

**Method:**

The records of 732 consecutive patients who had undergone the bypass were retrospectively analyzed and divided in two groups, group 1 with 12 mm anastomosis (n=374), and group 2 with 15 mm (n=358).

**Results:**

The groups showed anastomotic stenosis with rates of 11% and 3.1% respectively, with p=0.05. Other variables related to the anastomosis were also analyzed, but without statistical significance (p>0.05).

**Conclusion:**

The diameter of the anastomosis of 15 mm was related with lower incidence of stenosis. It was found that these patients had major bleeding postoperatively and lower surgical site infection, and in none was observed presence of anastomotic leak.

## RESUMO

**Racional:**

Entre as opções para o tratamento cirúrgico da obesidade, o mais utilizado é o bypass gástrico em Y-de-Roux. A anastomose gastrojejunal dele pode ser realizada de duas maneiras, manualmente ou utilizando grampeador linear e circular, e as complicações são dividas em precoces e tardias.

**Objetivo:**

Comparar a incidência de complicações precoces relacionadas com a confecção manual da anastomose gastrojejunal no bypass gástrico utilizando sonda de Fouchet com calibres diferentes.

**Métodos:**

Foi realizada análise retrospectiva transversal com 732 pacientes submetidos ao procedimento, divididos em dois grupos, grupo 1 com anastomose de 12 mm (n=374), e grupo 2 de 15 mm (n=358).

**Resultados:**

Os grupos apresentaram taxas de estenose de anastomose de 11% e 3,1% respectivamente, com p=0,05. Outras variáveis relacionadas à anastomose também foram analisadas, porém sem significância estatística (p>0,05).

**Conclusão:**

O diâmetro da anastomose de 15 mm esteve relacionado à menor ocorrência de estenoses. Verificou-se, contudo, que estes pacientes apresentaram maior sangramento no pós-operatório e menor infecção de sítio cirúrgico. Não ocorreram fístulas na presente casuística.

## INTRODUCTION

The prevalence of obesity in Brazil is increasing every year. This elevation and the association with the failure rate in clinical treatment is related to the increasing demand for bariatric surgery^1^ ^,^ ^2^. Surgical options for morbid obesity include Roux-en-Y gastric bypass (RYGB), gastric banding, vertical gastrectomy and biliopancreatic diversion. RYGB is the most performed procedure in Brazil and in the world^2^.

The gastrojejunal anastomosis RYGB can be performed in two ways: manually or using linear or circular stapler^3^. Complications related to bariatric surgery can be divided into early and late^4^. Early complications include fistulas, bleeding, intestinal obstruction and pulmonary embolism^5^. Late complications mainly include stenosis of gastrojejunostomy anastomosis^6^.

Stenosis occurs in 6-20% of patients undergoing the procedure, and the possible mechanisms for their formation include ischemia causing scarring, excessive scar formation and the perfoming anastomosis using staplers or manually^6^ ^,^ ^7^. The manually performed have lower rates of stenosis compared with the use of staplers^8^.

The aim of this study was to compare the incidence of complications related to the manual preparation of gastrojejunostomy using probe Fouchet with different calibers in patients undergoing RYGB.

## METHODS

Cross retrospective analysis was conducted with 732 patients who underwent RYGB in Bariatric Surgery and Metabolic Service of Holy House Hospital in Curitiba, Paraná, Brazil between January 2012 to March 2013.

No external restrictive materials, as a ring or band, were used. The functional gastric reservoir (pouch) had volume of 30 ml and trapezoidal shape after two rounds with linear cutting stapler 80 mm and rear reinforcement suture.

**FIGURE 1:**
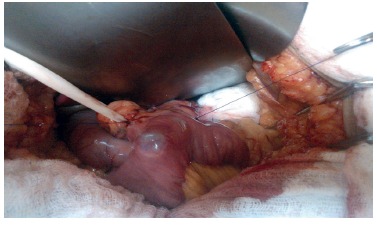
Final aspect of gastrojejunal laterolateral anastomosis with the gastric wall after the passage of the Fouchet probe for calibration

A food handle with 140 cm was placed in a pre-colic and pre-gastric position. Next, gastrojejunal anastomosis manual laterolateral was performed in anterior gastric wall in two layers with 3-0 absorbable monofilament long half-life in all patients (Figure 1).

Patients were divided into two groups: group 1 - 12 mm gastrojejunostomy anastomosis (Fouchet probe of 36 Fr), with 374 patients, and group 2 - 15 mm gastrojejunostomy anastomosis (Fouchet probe of 44 Fr), with 358 patients.

Data were collected on the incidence of various complications: presence of gastrojejunostomy anastomosis stenosis, occurrence of fistulas, bleeding with transfusion indication and surgical site infection.

## RESULTS

Both groups showed similar results with respect to age, gender, body mass index, presence of comorbidities such as hypertension, dyslipidemia, diabetes mellitus, sleep apnea and time of postoperative gastric bypass. 

Groups 1 (12 mm) with 374 patients and 2 (15mm) with 358 showed gastrojejunostomy stenosis rates of 11% and 3.1%, respectively, requiring dilatation. Statistical significance was verified with p=0.05. Other variables related to the anastomosis were also analyzed, but without statistical significance (p>0.05, Table 1).

**TABLE 1:** Incidence of complications related to gastrojejunostomy and gastric bypass

	Group 1 - n=374 (12 mm)	Group 2 - n=358 (15 mm)
Fistula occurrence	0,0%	0,0%
Postoperative bleeding	2,7% - n=10	4,7% - n=17
Anastomotic stenosis	11% - n=41	3,1% - n=11
Surgical site infection	2,1% - n=8	1,7% - n=6

## DISCUSSION 

Complications related to the RYGB include fistulas, postoperative bleeding, anastomotic stenosis and surgical site infection. The occurrence of post-RYGB anastomotic fistula varies from 0-6%, being more common appearance in the region just above the anastomosis. In this study the presence of fistula in the patients was not identified. Usually, the presence of fistula becomes necessary to perform a new surgical procedure to wash the abdominal cavity, drainage and placement of enteral feeding tubes. In patients with small fistula, clinical treatment can be considered^9^. 

Bleeding after surgery has an incidence between 1.9 to 4.4%, and may be higher in patients who have a history of previous abdominal surgery^10^. Among the patients studied, only group 1, with 12 mm gastrojejunostomy anastomosis, remained according to the rate reported in the literature, with an incidence of 2.8%, representing 10 of the 374 patients in the group, while in group 2, with 15 mm anastomosis, the occurrence of this complication was in 17 patients (4.7%), above the found in the literature. 

The postoperative bleeding can be originated in the gastric pouch, in the excluded stomach, in the food handle, in the gastrojejunal anastomosis and in the enteroentero anastomosis. The bleeding occurs at the edges of the severed tissue or in the tissue penetrated for the staplers, and the site of highest frequency is the line clip of the remaining stomach. Can be intraperitoneal or intraluminal, and prompt recognition is critical for good prognosis. However, as the abdominal wall of these patients is usually thick, the clinical signs are not lush, being able to lose large amounts of blood until the frame is clinically apparent^5^.

The bleeding with hemodynamic instability indicates the need for surgical intervention, while in stable patients expectant management can be adopted. In the early postoperative bleeding, until a few hours after the operation, with the presence of hematemesis or intestinal bleeding, it is indicated emergency surgery. However, in cases of late bleeding, more than 48 h after, can be adopt a conservative approach in most cases, when associated to the absence of active bleeding or hemodynamic instability^5^. 

The surgical approach can be performed by laparotomy or laparoscopic, with the laparoscopy contraindicated in cases of copious bleeding, for the possibility of worsening of symptoms as a result of the increased intraabdominal pressure. During operation, is performed the localization of bleeding, removal of clots and the strengthening of clipping lines^10^. If the bleeding have proximal and intraluminal origin, the best treatment is endoscopically. Some measures can be taken to reduce the risk of bleeding, as the use of smaller clips with loads 2.5 mm instead of 3.5 mm, realizing reinforcement suture lines stapling or the use of reinforcement products in the lines^5^.

Stenosis of the gastrojejunostomy occurs in 3-27 % of patients who underwent RYGB. Occurs on average 7.7 weeks after surgery, with the presence of nausea and postprandial vomiting, gastroesophageal reflux and partial or full dysphagia^6^ ^,^ ^11^. The use of linear staplers presents stenosis rate between 3.1 to 6.8 %^6^. As for the circular staplers varies with the diameter ^12^. The use of a circular stapler with 25 mm diameter have 6.2% incidence of stenosis, while the 21 mm diameter have 15.9%^13^.

Some systematic reviews and meta-analyzes reported that the stenosis rate occurs in a significantly higher number using a circular stapler compared to linear as well as increased operating. According to these studies, using circular or linear staplers do not influence the occurrence of fistulas, postoperative bleeding and marginal ulcers^14^.

The comparison of the anastomosis making with manual suturing and with the use of linear staplers, both 18 mm in diameter, the use of staplers has an incidence of 10.1% anastomotic stenosis, superior to manual suturing, with 4,1%. Regarding the presence of fistula or reoperation, there is no difference between the two techniques^8^. In the selected sample, it was found in group 2 (15 mm) 3.1% stenosis rate, lower than the 4.1% observed in the anastomosis with 18 mm diameter. In relation to group 1 (12 mm), the stenosis rate was higher, observed in 11%. Patients diagnosed with anastomotic stenosis were referred to endoscopic dilatation with pneumatic balloon.

The diagnosis is made clinically associated with additional tests, such as endoscopy or contrast radiography. Endoscopy is the method of choice due to its greater sensitivity^6^. The treatment of the stenosis is usually accomplished with the use of endoscopic dilatation with pneumatic balloon, with a resolution of 95 % and an average of 2.1 sessions, although there is not a well defined protocol for that type of situation^15^ ^,^ ^16^. In case of failure of the procedure, it is necessary surgical intervention in 0.05 % of cases^17^. The recurrence of the stenosis of the anastomosis after two dilations or fibrosis in gastrojejunal can be treated with sternotomy^11^.

Endoscopic dilation is not without complications, with a rate of 3%^17^. Furthermore, there is no consensus that the procedure, if performed early, is considered safe^18^. Perfuration of the gastrojejunostomy is the main complication, being the most patients conservatively treated ^19^. Complete resolution of the stenosis is not well established, because although the initial objective being the relief of symptoms, must be maintained narrow anastomosis to guarantee weight loss^20^. The use of a 15 mm diameter balloon is considered safe because does not affect weight loss and decreases the need PF a next dilating^21^.

The surgical site infection has an incidence of 8-15 % and may be superficial or affect the tissue more deeply^22^. The presence of this complication can also increase the risk of incisional hernia^23^. In both groups studied, the occurrence of surgical site infection remained below the incidence reported in the literature. Group 1 have rate of surgical site infection in eight patients (2.1%) and group 2 in seven (1.7%). For patients who perfomed RYGB, some factors may increase the risk of surgical site infection development, as a BMI greater than 50 kg/m², delayed prophylactic antibiotic administration, use of epidural anesthesia, presence of sleep apnea and time top surgical 180 min^22^.

## CONCLUSION

The 15 mm diameter anastomosis was related to a lower incidence of stenosis. However, it was found that these patients had major bleeding postoperatively and lower surgical site infection. There were no leaks in the present series.
